# Comparative study of impaction and sedimentation in an aerosol chamber using defined fungal spore and bacterial concentrations

**DOI:** 10.1371/journal.pone.0187039

**Published:** 2017-12-19

**Authors:** Doris Haas, Herbert Galler, Carola Fritz, Christina Hasler, Juliana Habib, Franz F. Reinthaler

**Affiliations:** Institute of Hygiene, Microbiology and Environmental Medicine, Department of Environmental Hygiene, ZWT Medical University of Graz, Neue Stiftingtalstraße 2, Graz, Austria; Universita degli Studi di Milano-Bicocca, ITALY

## Abstract

Biocontamination control is a very significant part of the manufacturing process of sterile drugs. Sterility is frequently monitored by active or passive air sampling measurements, but there are no specific rules as to how this is to be done. This study tested air sampling methods of active impaction and passive sedimentation under standardized conditions. *Aspergillus niger* (*A*. *niger*) and *Staphylococcus aureus* (*S*. *aureus*) were selected in this experiment to examine parallels, correlations and differences between the two methods. The results show that the number of colony forming units per plate (CFU/plate) was higher for *A*. *niger* in the active method, whereas for *S*. *aureus* it was higher in the sedimentation method. A high correlation coefficient was found between the impaction and sedimentation methods for *A*. *niger*. For *S*. *aureus*, depending on the culture media used and the time for passive air sampling, a larger number of CFU/plate was found than in active air sampling. This study concludes that active and passive air sampling can be used for monitoring the air in clean rooms. For fungal spore detection, the impaction is more efficient, as it is possible to sample a higher volume of air in a shorter period of time, whereas the optimal measurement methods for *S*. *aureus* depend on a number of factors.

## Introduction

In practice, different methods of measuring airborne microorganisms have proven successful. Airborne microorganisms can be measured with different procedures that can be classified as active and passive. Two methods commonly used are impaction sampling (active process) and passive sedimentation where the microorganisms settle directly on the agar media [1, 2]. For qualitative and quantitative determination of microorganisms indoors and outdoors, an active impactor is used to assess the degree of contamination [3–5]. The impaction method uses a defined volume of air with germinal material which impacts on agar inside the device.

Special procedures should be applied in industrial microbiology and clinical diagnostics in order to keep the risk of biological contamination to a minimum. This requires sedimentation plates which are openly positioned in the working space during and/or after work. The EU-Guidelines for Good Manufacturing Practice set limits for the microbiological contamination with regard to clean room classification [6] (EU-GMP, 2008). Gutschi found that the results of active measurements of airborne microorganisms were more constant and higher using the Microbial Air Sampler MAS-100NT® than those using the passive sedimentation method. The MAS-100 NT® measures the microorganisms in clean rooms, sterile environments in pharmaceutical companies and other highly sensitive areas. Thus, the active measuring method should be preferred over the passive one for contamination control in the production of sterile drugs. Moreover, this experiment demonstrated a non-significant correlation of the measuring procedures [7]. Canha et al. found a correlation between the active and the passive methods which were used to study indoor and outdoor air under normal conditions [8]. According to Petti et al., the active measuring method is more reliable at lower levels of contamination. For higher concentrations of microorganisms, the passive method was used, which is less expensive than the active method [9]. Napoli et al. found that the two methods correlate in a similar manner with the air quality [10]. Whyte, however, holds that the passive method is more suitable for monitoring concentrations of airborne microorganisms in drug manufacturing [11]. Montacutelli et al. found that the active method yielded smaller concentrations of microorganisms than the passive method, both with regard to bacteria and to fungi [12]. The investigations by Asefa et al. and Verhoeff et al. showed that the active impaction method for quantitative evaluation was more efficient than the sedimentation method. Qualitatively, the two methods showed similar results [13, 14].

These very different findings led to the development of an experiment to test both the active and the passive measuring methods under standardized conditions and to investigate whether the results show a correlation.

The first goal of this study was to find a suitable, standardized method of measurement in order to be able to investigate defined fungal spore and bacterial concentrations in an aerosol chamber using active and passive methods. The active method used impaction, the passive sedimentation. The second goal was to analyze the correlation between the two methods through the linearity of measurement data as well as through the recovery rate of spore concentrations in the aerosol chamber under standardized conditions. For this study, *Aspergillus niger* (*A*. *niger*) and *Staphylococcus aureus* (*S*. *aureus*) were selected and investigated in different concentrations in order to demonstrate correlation, parallels and differences between the two methods. With these results, a further evaluation of these very different measuring methods (active and passive) would become possible.

## Experimental procedures

### Description of the aerosol chamber

**Fig 1:**
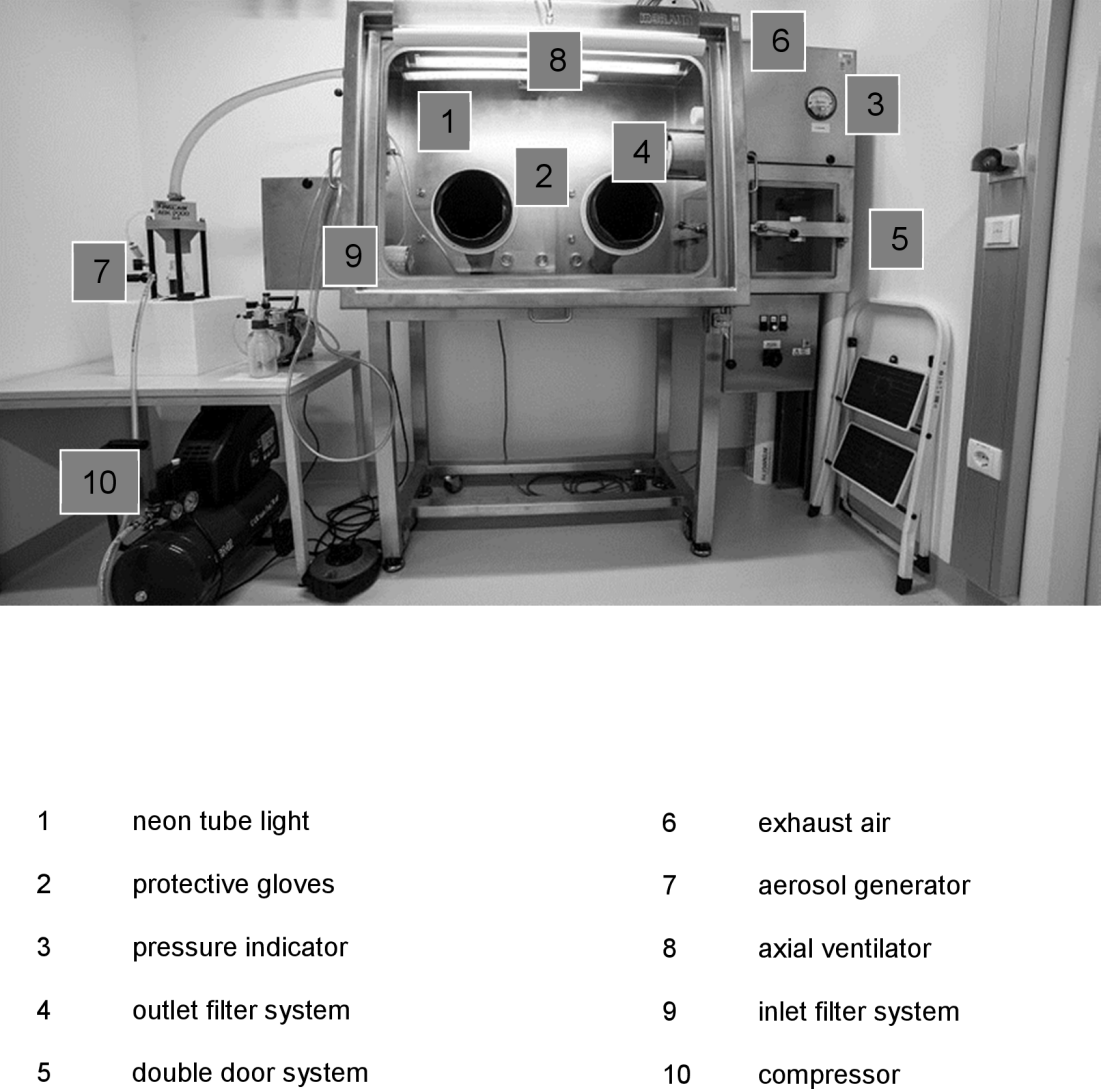
Design of ISO-PRO-under pressure isolator (MECALAB/MBraun® AG).

The ISO-PRO-under-pressure Isolator (MECALAB/MBraun AG, Germany) consists of an aerosol chamber and a transfer chamber with a double door system which serves to load and unload growth media and the air sampler MAS-100NT® (http://www.mbv.ch/documents/flyer_mas100_nt_1.pdf. Last access: 26 July 2016). It is equipped with protective inlet and outlet filters. A ventilation system on the right hand inside wall with a high efficiency particulate air (HEPA) filter classification H13 sucks the air out of the chamber again. In addition, a constant maximum negative pressure of 300 Pa is generated. There is a axial ventilator on the ceiling of the aerosol chamber and in the upper front area there is a neon tube lighting the work space. Both ventilator and fan can be switched on individually. The aerosol chamber is connected to the aerosol generator (PALAS® AGK 2000) by means of a tube system. With the aerosol generator, solid particle aerosols can be generated from suspensions. The suspension medium for both aerosols tested was 0.85% NaCl to help maintain cell integrity and viability, and Tween80 (0.01%) was added for the aerosols containing fungi. A nozzle specially developed by Palas® prevents crystallization of salt at the nozzle outlet, thereby facilitating atomization of salt solutions with high dosing consistency. The flow rate of the aerosol generator is 3–10 L/min http://www.palas.de/de/product/agk2000 (Fig 1).

### Sampling methods

Active sampling was carried out by a one-stage microbial air sampler MAS-100NT® operating at a flow rate of 100 L/min [15]. The cut-off-size of this device (d_50_ = 1.7 μm) is adapted to collect airborne microorganisms with an aerodynamic diameter of ≥ 2 μm [16]. A disinfecting agent was used for cleaning the device after each sampling.

In passive sampling, using the sedimentation method, also called “open-dish method“, the petri dishes were placed centrally on the bottom of the aerosol chamber. Following a defined aerosol flow time, the plates were opened for the microorganisms to settle.

For measurements and evaluations of the fungal spores, Malt Extract Agar (MEA) and Dichloran Glycerol Agar (DG18) were incubated at 37°C for 24–48 h. Testing of the bacterial strains was carried out using Casein peptone Soybean flour peptone Agar (CASO) and Colistin Nalidixic Acid Agar with 5% sheep blood (CNA) at 37°C each for 24h. The relative humidity and air temperature were measured with the infrared thermo hygrometer testo®445 (Testo GmbH, Austria) while conducting the test series in the chamber.

### Preliminary experiments

For the standardized test series, suitable measuring parameters were tested in the preliminary experiment:

▪ Definition of bioaerosol concentration of the suspension
▪ Operation of aerosol chamber with and without fan
▪ Selection of different sampling times of up to 24h
▪ Flow time for filling the aerosol chamber
▪ Air sampler MAS-100NT® with different sampling volumes
▪ Sedimentation with different plate exposure times
▪ Position of sampler and growth media in aerosol chamber
▪ Monitoring of stability of suspensions

Fungal species *A*. *niger* (ATCC®16404) and bacterial strain *S*. *aureus* (DSM 799) were selected as test strains. These microorganisms are particularly important for the health of humans and can easily be detected in aerosols.

For the production of *A*. *niger* stock solution, a pure culture was initially cultured on Malt Extract Agar and incubated at 37°C. Following an incubation period of 24 to 48h, a randomly selected portion of spores was suspended in 100 ml NaCl (0.85%) + Tween80 (0.01%) and filtered through a sterile gauze bandage. The CRYO-pellets loaded with bacterial test strain S. *aureus* were placed in 100 ml sterile distilled water and subsequently shaken for 30 min using the SKI 4 Shaking incubator.

For the determination of the *A*. *niger* concentration, 100 μl of the stock solution were collected and dilutions of 10^−1^ to 10^−6^ prepared. From each of these solutions, 100 μl were plated in duplicate on malt plates and incubated at 37°C. In order to be able to determine the concentrations of the bacterial suspensions, the samples were also plated in duplicate in different dilutions (10^−1^–10^−3^) onto the culture media CASO and CNA and incubated at 37°C. Following the incubation period, the colony forming units (CFU) were counted and the fungal spore and bacterial concentrations were determined in CFU/ml respectively.

**Fig 2:**
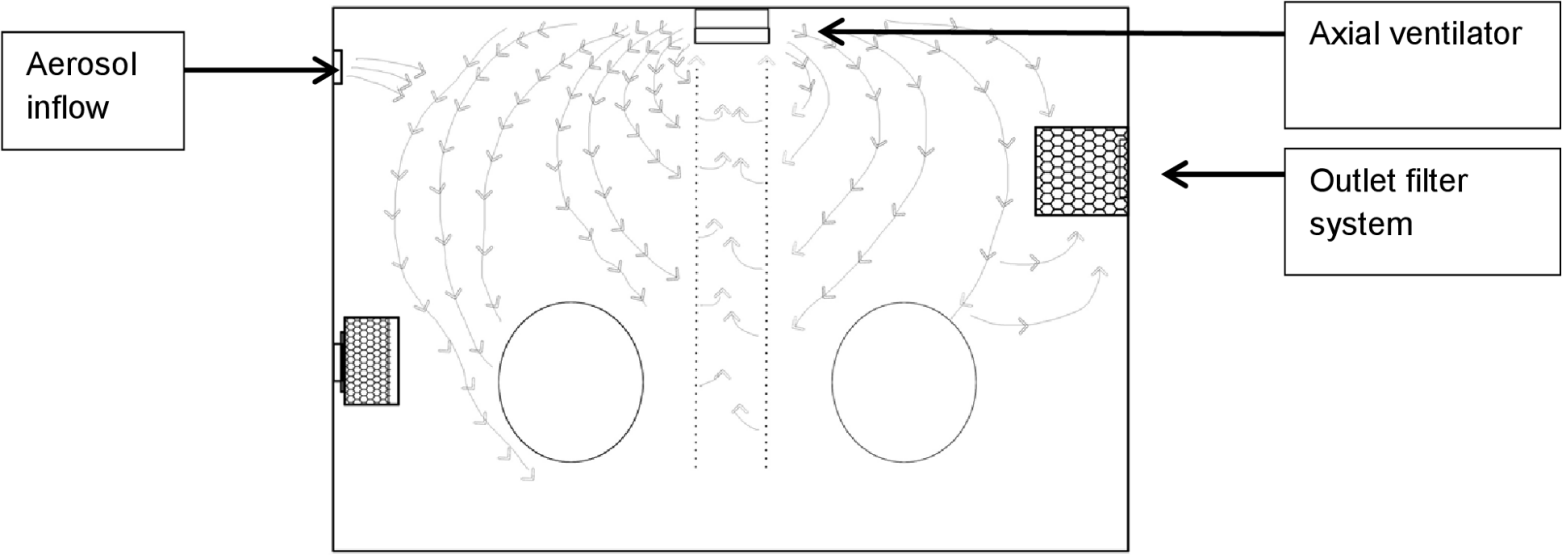
Air circulation in the aerosol chamber.

In order to find a suitable standardized procedure in active and passive sampling, a series of preliminary experiments was conducted. First, the conditions for the different measurements with the MAS-100NT® and the sedimentation method inside the aerosol chamber were changed by alternately operating and not operating the outlet filter system and/or axial ventilator (Fig 2) and comparing the obtained results. Then the frequency and time intervals of the active and passive airborne microorganism measurements were modified after aerosol inflow. The active and passive measurements of fungal spores and bacteria were conducted prior to the aerosol inflow and after 2, 3, 6, 10, 15, 20, 30, 60 and 120 min of aerosol inflow. For the preliminary tests, the suspensions with defined fungal spore and bacterial concentrations (CFU/ml), which were in the optimal countable range, were used for the standardized test series. One hour of aerosol flow was needed to fill an aerosol chamber with a volume of 0.65 m^3^.

The stability of fungal and bacterial suspensions were monitored in each measuring series at the beginning, after one hour and after two hours by plating 1 μl and 10 μl onto the growth media in duplicate and incubating for 24 hours at 37°C prior to counting.

### Standardized test series with impaction and sedimentation methods

Based on the results obtained from the preliminary tests, a procedure was selected which included 30 measurements of both actively and passively collected airborne microorganisms.

The concentrations of the suspensions most suitable for the measurements were between 10^2^ CFU/ml and 10^4^ CFU/ml for the fungal spores and between 10^3^ CFU/ml and 10^5^ CFU/ml for bacteria. From each duplicate determination on the culture media used, the mean value was calculated for the determination of CFU/ml.

In the standardized test series, both measuring methods were conducted without operating either outlet filter system or axial ventilator. Prior to measurements, the first stability monitoring of the suspension was carried out and the aerosol chamber was cleaned by a 10 min rinse with distilled water combined with the outlet filter system for suctioning off the air and disinfected to be sure that it was free of contaminations. Subsequently, 100 ml of the standard suspension were filled into the designated vessel of the aerosol generator. This required defined fungal spore and bacterial concentrations which were monitored through stability controls. Moreover, the aerosol inflow was started exactly 1 hour prior to the measurements in order to fill the chamber.

Before the start of aerosol flow, growth media and the air sampler were brought into the chamber by way of the transfer chamber and positioned in the center. Following an aerosol flow of one hour, the second stability monitoring was carried out; subsequently the active aerosol measurements were started. All measurements were carried out in duplicate. For the active measurements of airborne microorganisms, the air sampler MAS-100NT® with a sampling volume of 50 L/30 sec for *A*. *niger* and 100 L/min for *S*. *aureus* was selected in the preliminary experiment. For *A*. *niger*, higher sampling volumes led to overlapping of the fungal colonies on the agar plate which made colony counting difficult.

Following the removal of the air sampler and the growth media, the aerosol chamber was again cleaned as mentioned above, followed by renewed aerosol flow for one hour. In the course of the subsequent passive measurements, 4 MEA and 4 DG18 plates were opened and exposed to *A*. *niger* for 1 h; two petri dishes of CASO and two of CNA were exposed to *S*. *aureus* for 1 h and for 2 h. Immediately after 1 hour flow, stability of the test suspension was monitored for the third time. Then the culture media were incubated, the CFU/plate (sedimentation) and CFU/ml (test suspension) were counted.

### Statistical evaluations

MS Excel was used to determine the linear relationship, correlation coefficient and the proportion as well as microbial recovery between impaction and sedimentation. The coefficient of determination (R^2^) expresses the linear relationship between two variables. R^2^ can take on a value between 0 and 1, a value close to +1 expressing a linear relationship between the two variables. The correlation coefficient (r) represents a dimensionless measure, which can be used for judging the degree of the linear relationship between two interval-scaled variables. Possible values for the correlation coefficient lie between -1 and +1. A value close to +1 shows a linear relationship; a correlation coefficient of 0 shows no linear relationship. For the calculation of the ratio between active and passive measuring methods, the results (CFU/plate) of the impaction were divided by those of the sedimentation method for each measurement of the 30 test series; the average was calculated and evaluated separately for the two growth media.

To avoid a bias in starting concentration of microorganisms and the repeated measurements of active and passive methods, statistical corrections were carried out (S1 Fig).

## Results

The mean values of the 30 measurements arranged in ascending order according to the standardized concentrations of test strains in the suspension are listed in Tables 1 and 2. The standardized concentrations of *A*. *niger* were between 1.07x10^2^ CFU/ml and 2.05x10^4^ CFU/ml and of *S*. *aureus* between 4.61x10^3^ CFU/ml and 1.84x10^5^ CFU/ml. In three bacteriological measurements, no active measurements could be carried out due to a technical problem and no values were specified.

**Table 1:** Impaction measurements (n = 30) of *A*. *niger* and *S*. *aureus* with mean values in CFU/ml (test suspension) and CFU/plate.

Impaction	test series n = -30				
*A*. *niger* CFU/ml	CFU/plate MEA	CFU/plate DG18	*S*. *aureus* CFU/ml	CFU/plate CASO	CFU/plate CNA
1,07E+02	6	6	4,61E+03	5	6
2,61E+02	9	5	6,42E+03	11	15
3,33E+02	22	20	9,93E+03	10	27
4,33E+02	4	2	1,29E+04	27	40
4,56E+02	24	25	1,41E+04	17	25
1,06E+03	44	39	1,51E+04	24	30
1,19E+03	36	30	1,53E+04	27	33
1,40E+03	32	41	1,82E+04	51	101
1,53E+03	68	57	2,00E+04	n.s.	n.s.
1,63E+03	72	64	2,53E+04	77	63
2,00E+03	97	103	2,53E+04	48	81
2,07E+03	112	106	2,84E+04	63	108
2,25E+03	82	67	2,84E+04	63	73
2,30E+03	113	121	2,99E+04	59	90
2,36E+03	106	100	3,13E+04	101	202
3,42E+03	139	128	3,13E+04	51	70
3,68E+03	117	114	3,67E+04	101	120
3,69E+03	240	278	3,47E+04	n.s.	n.s.
3,89E+03	132	164	4,60E+04	72	79
3,91E+03	234	246	4,77E+04	100	140
4,08E+03	223	207	4,82E+04	92	182
5,07E+03	207	164	5,01E+04	n.s.	n.s.
5,63E+03	162	141	5,12E+04	178	192
8,55E+03	269	250	7,32E+04	128	191
9,19E+03	314	288	7,69E+04	207	216
9,19E+03	387	314	7,95E+04	188	274
1,14E+04	312	276	9,37E+04	561	356
1,17E+04	279	260	1,26E+05	536	566
1,79E+04	325	316	1,30E+05	138	307
2,05E+04	408	342	1,84E+05	249	356

n.s.: not specified

**Table 2:** Sedimentation measurements (n = 30) for *A*. *niger* and *S*. *aureus* with means in CFU/ml (test suspension) and CFU/plate.

Sedimentation test series n = 30							
	exposure time 1h			exposure time 1h		exposure time 2h	
*A*. *niger* CFU/ml	CFU/plate MEA	CFU/plate DG18	*S*. *aureus* CFU/ml	CFU/plate CASO	CFU/plate CNA	CFU/plate CASO	CFU/plate CNA
1,07E+02	1	0	4,61E+03	12	6	11	7
2,61E+02	1	0	6,42E+03	23	23	59	63
3,33E+02	2	3	9,93E+03	33	11	93	44
4,33E+02	0	1	1,29E+04	31	26	70	41
4,56E+02	1	2	1,41E+04	71	24	136	60
1,06E+03	5	4	1,51E+04	33	36	75	64
1,19E+03	5	3	1,53E+04	67	46	110	119
1,40E+03	5	4	1,82E+04	111	66	189	128
1,53E+03	5	5	2,00E+04	78	49	160	83
1,63E+03	4	3	2,53E+04	60	45	229	71
2,00E+03	8	8	2,53E+04	87	49	248	72
2,07E+03	12	11	2,84E+04	166	100	233	159
2,25E+03	10	7	2,84E+04	127	62	266	176
2,30E+03	10	11	2,99E+04	58	54	123	107
2,36E+03	18	20	3,13E+04	145	93	357	167
3,42E+03	21	17	3,13E+04	65	73	217	109
3,68E+03	10	9	3,67E+04	205	112	332	132
3,69E+03	37	27	3,47E+04	224	65	319	201
3,89E+03	16	14	4,60E+04	252	120	367	157
3,91E+03	26	22	4,77E+04	223	155	411	211
4,08E+03	24	21	4,82E+04	694	250	522	528
5,07E+03	22	27	5,01E+04	262	118	655	194
5,63E+03	22	23	5,12E+04	186	115	301	207
8,55E+03	40	28	7,32E+04	602	422	604	598
9,19E+03	64	53	7,69E+04	333	239	636	464
9,19E+03	64	58	7,95E+04	176	115	438	299
1,14E+04	63	53	9,37E+04	364	347	534	624
1,17E+04	38	40	1,26E+05	562	352	1054	718
1,79E+04	58	48	1,30E+05	762	222	n.c.	n.c.
2,05E+04	103	84	1,84E+05	998	952	n.c.	n.c.

n.c.: not countable

### Comparison of the two agar media

**Fig 3:**
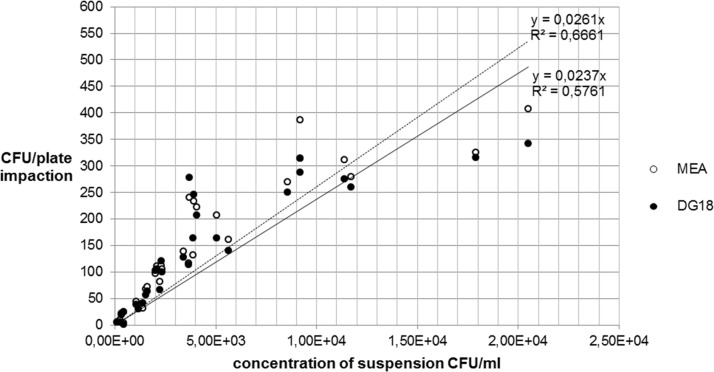
Comparison between MEA and DG18-agar impaction for *A*. *niger*.

**Fig 4:**
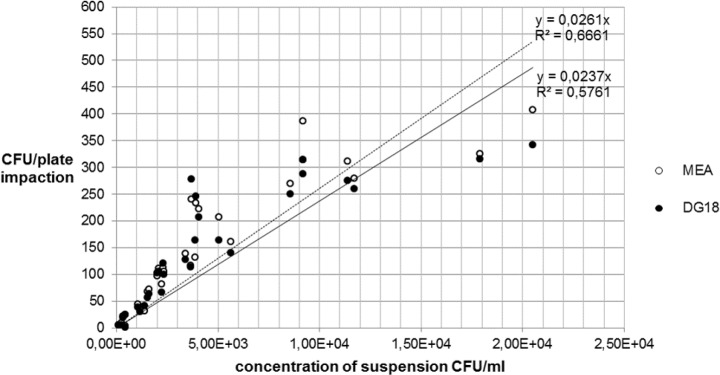
Comparison between CASO- and CNA-agar impaction for *S*. *aureus*.

**Fig 5:**
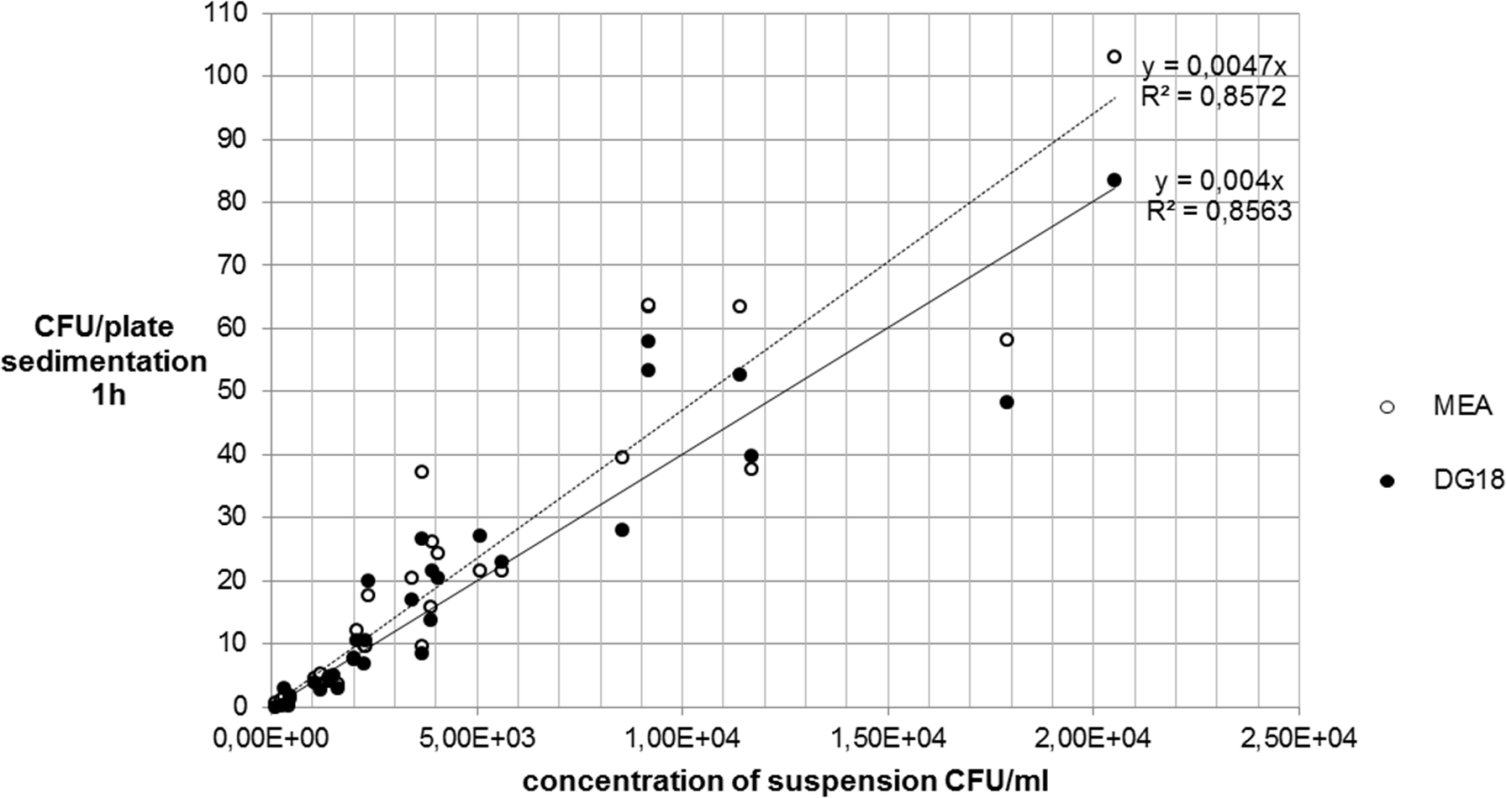
Comparison between MEA and DG18 agar sedimentation 1h for *A*. *niger*.

**Fig 6:**
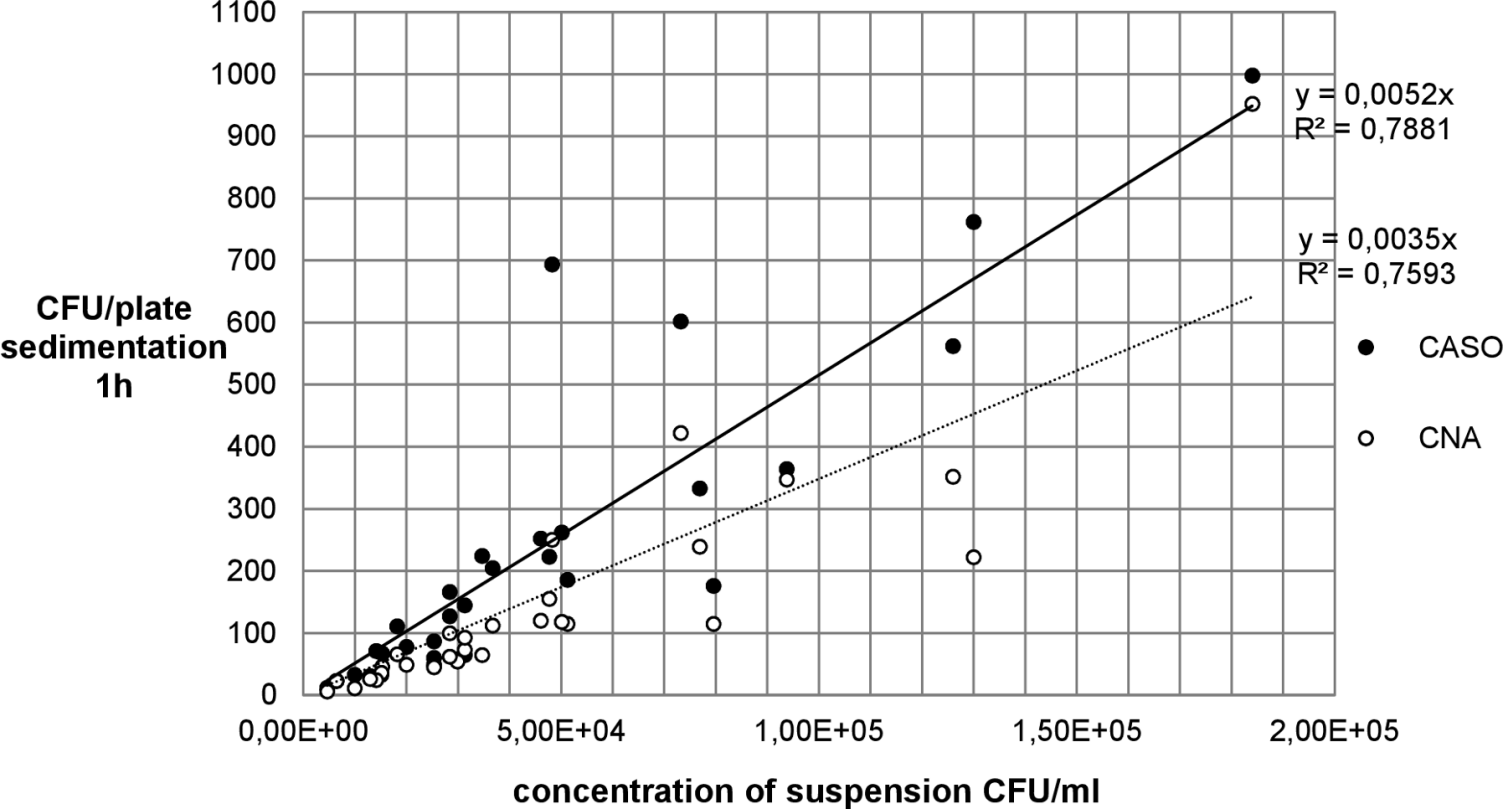
Comparison between CASO- and CNA-agar sedimentation 1h for *S*. *aureus*.

The colony forming units per plate (CFU/plate) of the fungal (MEA- and DG18) and the bacterial strains (CASO und CNA) in the impaction and in the sedimentation measurements were counted and the mean values recorded in Tables 1 and 2, and presented graphically (Figs 3–6).

#### Impaction method

In the impaction method, a comparison between the two growth media shows no significant difference for either of the tested strains.

The numbers of *A*. *niger* colonies ranged between 6 and 408 CFU/plate for MEA, and between 6 and 342 CFU/plate for DG18. The numbers of *S*. *aureus* colonies were between 5 and 561 CFU/plate for CASO-agar and between 6 and 566 CFU/plate for CNA-agar. While the number of CFU/plate of fungi using impaction were continuously in the three-digit range starting at a concentration of 2.30x10^3^ CFU/ml, three-digit values for bacteria only started at a concentration of 5.12x10^4^ CFU/ml (Table 1).

Fig 3 shows that the two growth media yielded very similar coefficients of determination (R^2^) for *A*. *niger*, whereas in Fig 4 CNA shows a higher R^2^ than CASO for *S*. *aureus*.

#### Sedimentation method

The comparison between the two growth media in the sedimentation method shows no significant difference in the CFU/plate for *A*. *niger* (Table 2). The values were between 1.0x10^0^ and 10.0x10^2^ CFU/plate. At higher concentrations of the test solution the number of CFU on MEA is slightly higher than the number of CFU on DG18 agar; lower spore concentrations yielded almost the same results. For *S*. *aureus*, almost all plates in the passive sampling method yielded more CFU/plate than in active sampling. The colony forming units were between 12 and 998 CFU/plate for CASO-agar and between 6 and 952 CFU/plate for CNA-agar, respectively. Also, there are different results in the number of CFU/plate between 1 h and 2 h of sedimentation. On almost all plates which were exposed for 2 h, the three-digit range of CFU/plate started at a concentration of 1.53x10^4^ CFU/ml. At very high concentrations of the test strain in the aerosol starting at 1.30x10^5^ CFU/ml, the bacterial plates could no longer be counted.

Figs 5 and 6 show that the two growth media used yielded similar results (R^2^) both for *A*. *niger* and for *S*. *aureus*.

### Comparison of impaction and sedimentation

#### Linear relationship, coefficient of determination and correlation coefficient

In this study, the active and passive methods were contrasted using known concentrations of microorganisms in the individual measurements. The correlation between impaction and sedimentation was calculated and R^2^ was determined. In the case of *A*. *niger*, the coefficient of determination for MEA is at R^2^ = 0.8574 (S2 Fig) and for DG18 agar at R^2^ = 0.805 (S3 Fig). This means that there is very good relationship between the two measuring methods and thus a high correlation. For *S*. *aureus*, there were low coefficients of determination between 1 h sedimentation and impaction, namely R^2^ = 0.1455 for CASO-agar and R^2^ = 0.4807 for CNA-agar. This suggests deviations of single measurements from the linear trend line. Higher R^2^ values were reached between 2 h sedimentation and impaction: R^2^ = 0.3774 for CASO-agar and R^2^ = 0.7367 for CNA-agar (S4 Fig).

Table 3 shows the calculated correlation coefficients (r) between impaction and sedimentation. The values show a high correlation between the two methods for *A*. *niger* and after 2 h sedimentation for *S*. *aureus*.

**Table 3:** Correlation coefficient (r) of impaction and sedimentation for *A*. *niger* and *S*. *aureus*.

**Correlation (r) *A*. *niger***	**MEA**	**DG18 agar**
Impaction: Sedimentation 1 h	0.94	0.91
**Correlation (r) *S*. *aureus***	**CASO-agar**	**CNA-agar**
Impaction: Sedimentation 1 h	0.52	0.69
Impaction: Sedimentation 2 h	0.80	0.86
Sedimentation 1 h: Sedimentation 2 h	0.84	0.96

#### Recovery of microbial concentrations between impaction and sedimentation

**Fig 7:**
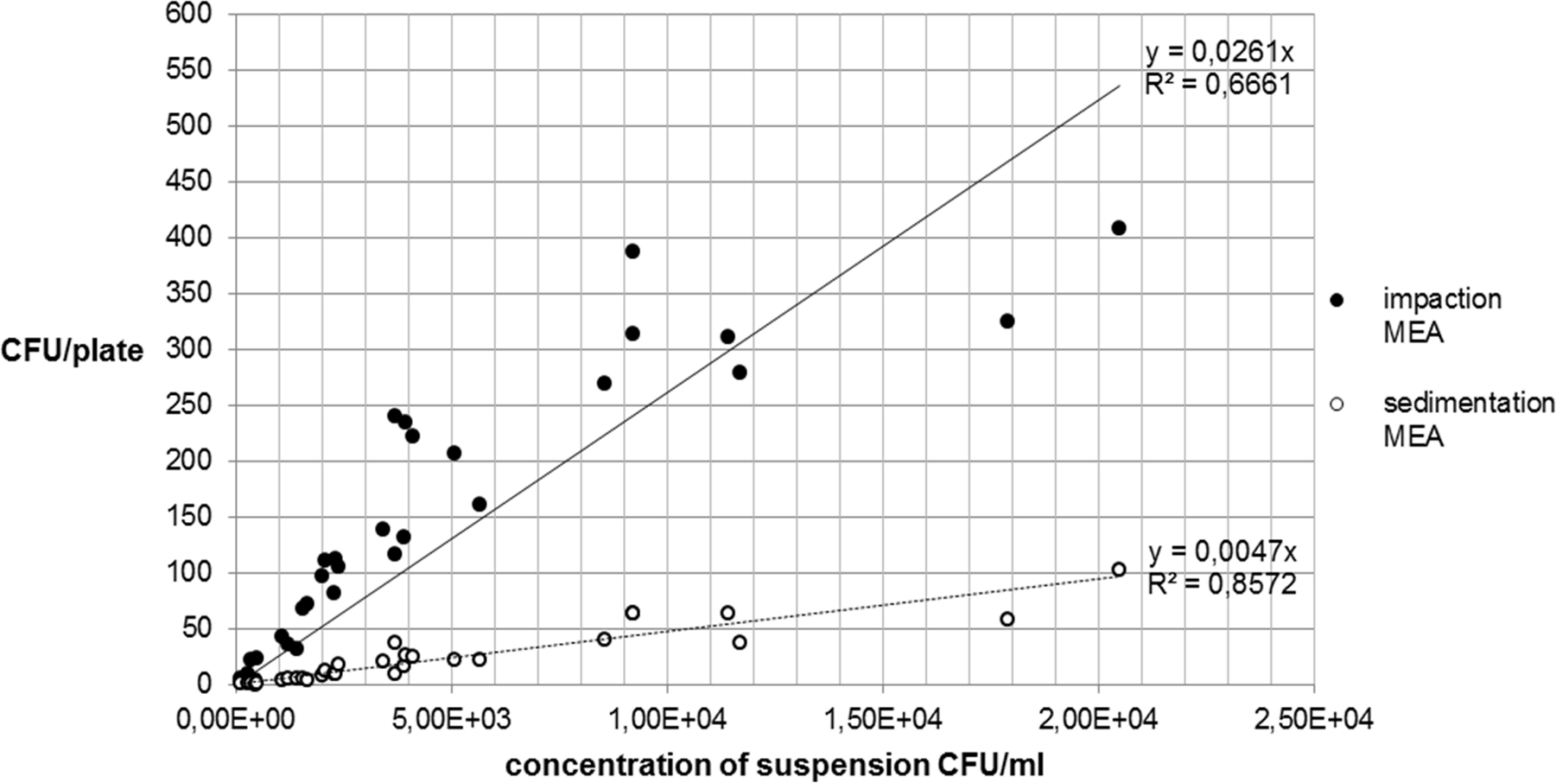
Recovery of *A*. *niger* concentration on MEA.

**Fig 8:**
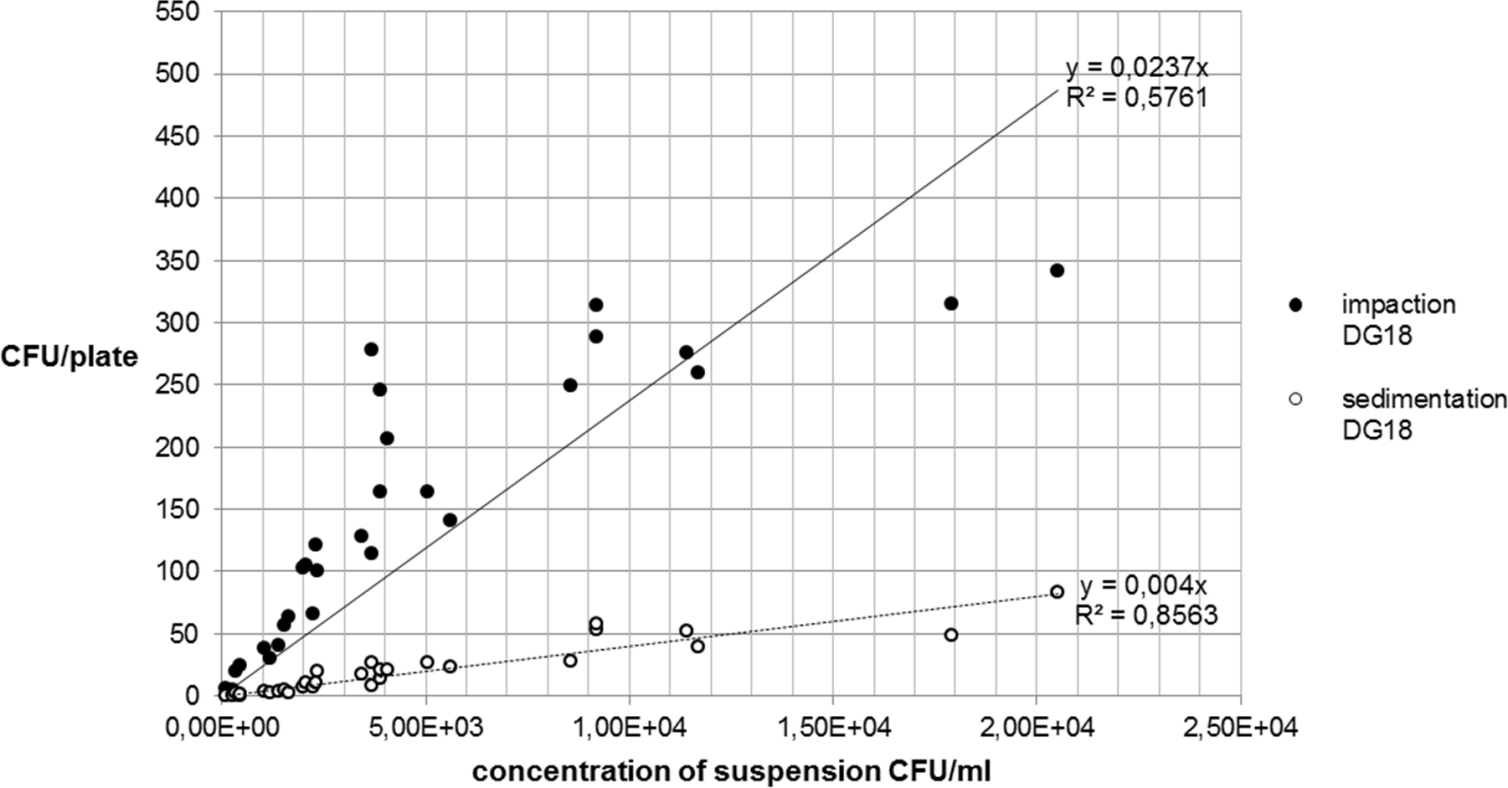
Recovery of *A*. *niger* concentration on DG18 agar.

The following Figs 7 and 8 show the relationship between the recovery of microbial concentrations of impaction, sedimentation and growth media.

The comparison between Figs 7 and 8 for *A*. *niger* shows that there are only small differences between the two growth media MEA and DG18. With regard to the fungal spore recovery in this standardized test series (CFU/plate), a considerable difference was found between the two methods.

**Fig 9:**
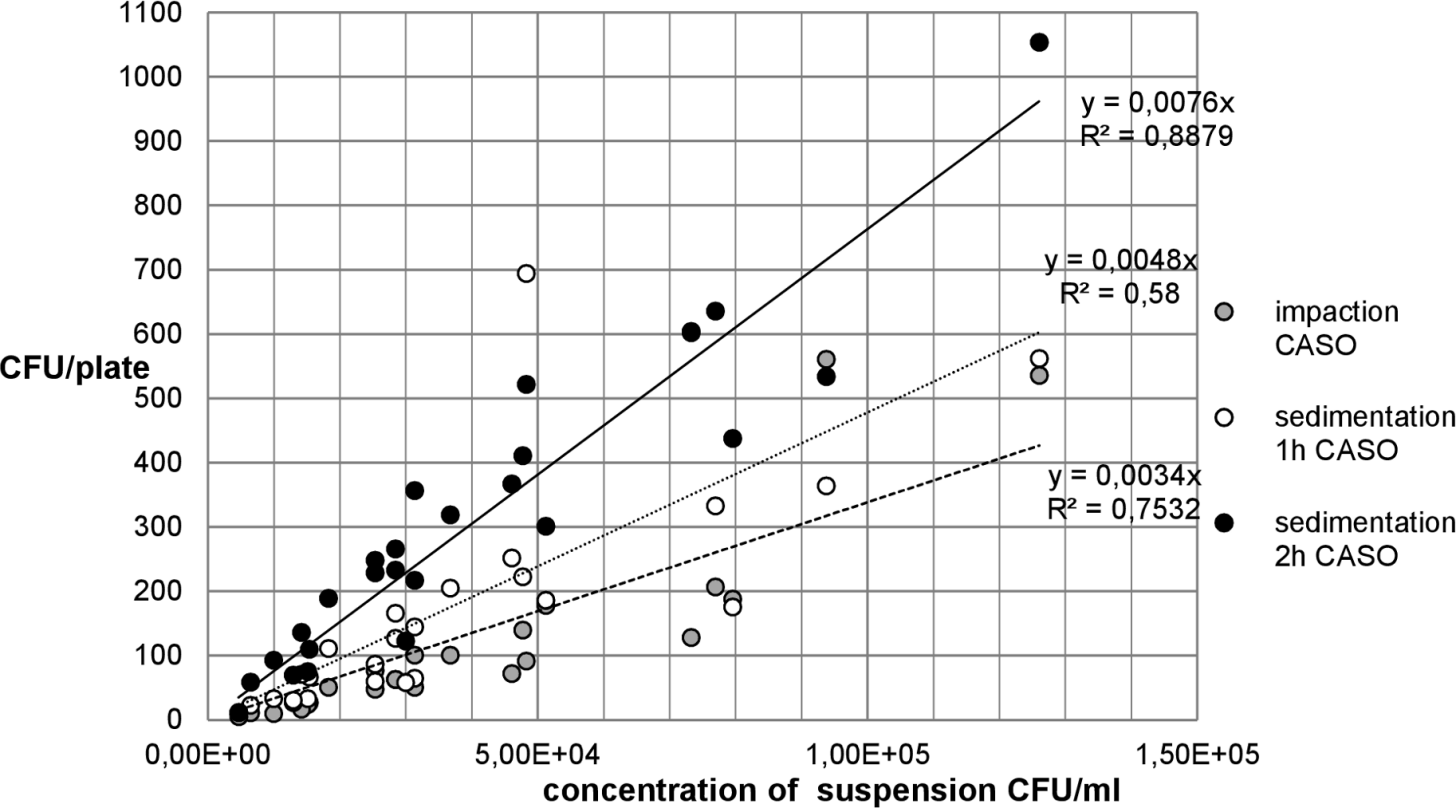
Recovery of *S*. *aureus* concentration on CASO-agar.

Fig 9 shows that the recovery of *S*. *aureus* colonies on CASO-agar was higher in passive than in active sampling. Up to a concentration of approximately 7.50x10^4^, the numbers of CFU/plate counted in both methods resemble each other and are close to the linear trend line. Higher concentrations show great fluctuations in the results, especially in the measurements using the MAS-100NT®.

**Fig 10:**
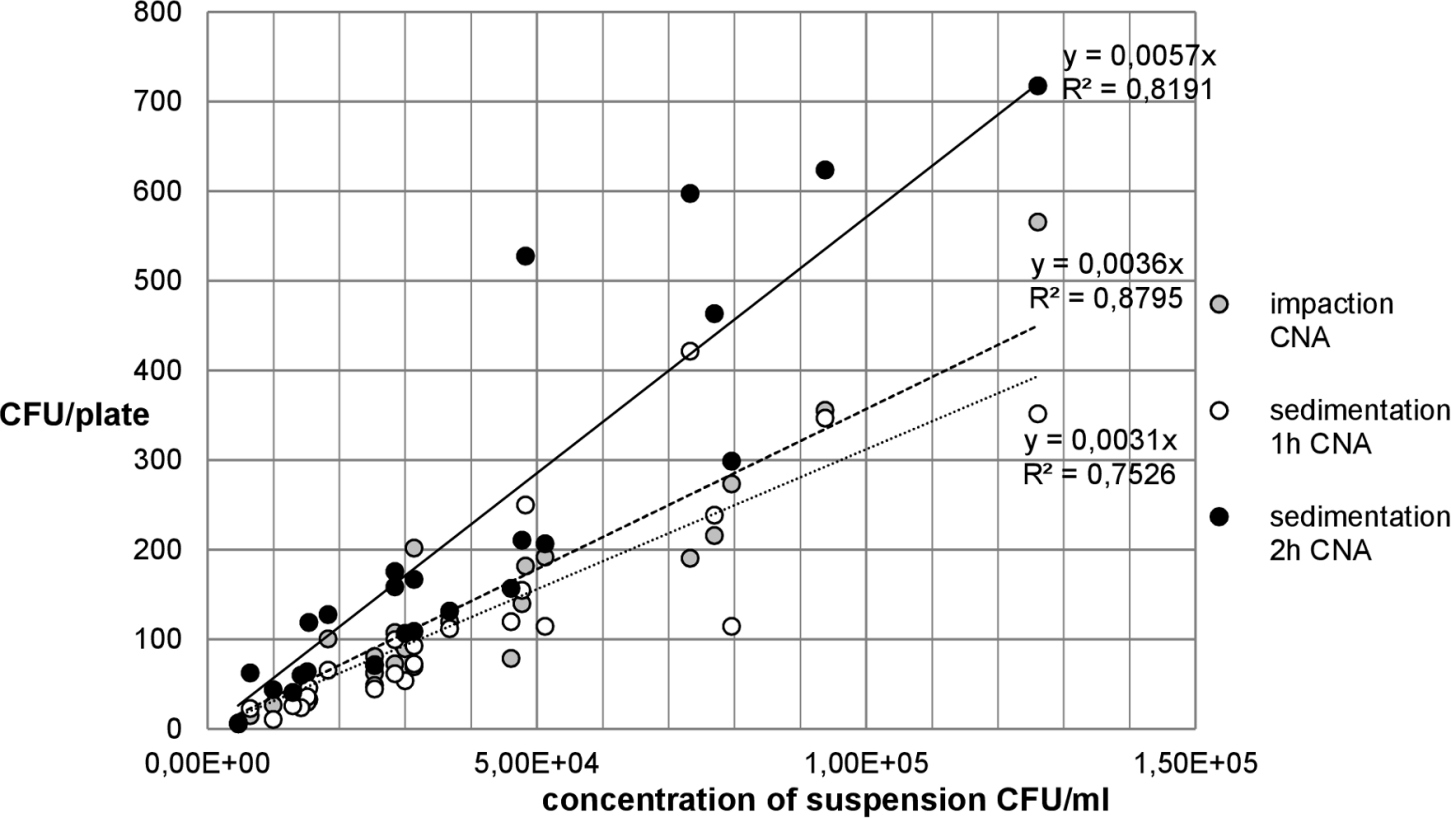
Recovery of *S*. *aureus* concentration on CNA-agar.

Fig 10 shows that at low *S*. *aureus* concentrations of up to approx. 5.00x10^4^ CFU/ml, the difference in the recovery of CFU/plate on CNA-agar in active and passive measurements with sedimentation of 1 h and 2 h is small. At higher concentrations, fluctuations and greater deviations in the number of CFU/plate from the trend line and between the different measuring methods occur. Moreover, the recovery of bacteria on CNA-agar is somewhat higher in impaction method than in sedimentation of 1 h.

#### Ratio between impaction and sedimentation

Between 1 h sedimentation and impaction, ratios of 1:9.4 and 1:9.6 were calculated for *A*. *niger*.

The ratio between the active and the passive method shows that for *S*. *aureus* 2.34 and 3.79 times as many CFU were counted in the 1 h and 2 h sedimentations as in the impaction on the CASO-agar. The numbers of CFU/plate also differ significantly depending on the sedimentation time. In collecting airborne microorganisms with the passive method, 1.96 times more loaded particles settled on the agar after a period of 2 h than after 1 h (Table 4).

**Table 4:** Ratio of impaction and sedimentation for *A*. *niger* and *S*. *aureus*.

**Ratio *A*. *niger***	**MEA**	**DG18 agar**
Sedimentation 1 h: Impaction	1: 9.4	1: 9.6
**Ratio *S*. *aureus***	**CASO-agar**	**CNA-agar**
Impaction: Sedimentation 1 h	1: 2.34	1: 0.97
Impaction: Sedimentation 2 h	1: 3.79	1: 1.75
Sedimentation 1 h: Sedimentation 2 h	1: 1.96	1: 1.91

A ratio of 1:1.91 between sedimentation times of 1 h and 2 h means that almost twice as many CFUs were on the CNA plates when the plates in the aerosol chamber were exposed for 2 hours. In contrast to the impaction method, sedimentation of 2 h yielded 1.75 times as many microorganisms. The ratio between impaction and sedimentation of 1 h was 1:0.97 (Table 4). In the active and the passive methods, the numbers of CFU/plate counted were almost identical, sometimes even higher on CNA plates with active measurements (Tables 1 and 2).

## Discussion

For biocontamination monitoring and ensuring sterility in special work processes, the current investigation tested both active measurement of airborne microorganisms using impaction and the passive method of sedimentation. Under standardized conditions, parallels, correlations and differences were analyzed. In order to standardize the sampling process, preliminary experiments tested the measuring parameters in the aerosol chamber. So far, many measuring systems and methods of analysis have been conducted in aerosol chambers in order to evaluate the survival rate of airborne microorganisms [17–19]. The aerosol chamber in the present study has different functions which were all tested in preliminary experiments in order to determine which settings are most suitable for the standardized test series. When looking at the results of the different methods of measurement, it soon became apparent that the bacterial counts changed with increasing aerosol flow time in the chamber if the outlet filter system and axial ventilator were in operation during measurement. If, for example, aerosol flow was interrupted, the microorganism concentration declined during operation of the ventilator, while microorganisms continued to be present in the chamber for a while during operation of the outlet filter system. According to Osborne et al., in the routine investigations and for biocontamination monitoring the ventilator and outlet filter system are used [20]. Pasquarella et al. observed that a wide variation of microbial contamination during surgical activity in operating theatres using similar forms of ventilation affected the air quality. In the present study, operating axial ventilator or outlet filter system made it impossible to create standardized conditions. Also, the aim was to create no additional or artificial air flow, so that the natural sedimentation of the loaded particles would not be affected [21]. The air turbulences may decrease the correlation between the active and the passive methods. Settled microorganisms can be stirred up from the ground, re-enter the air and be gathered by the Microbial Air Sampler [7]. Many studies report that stirring up microorganisms brings them back into the air and thus distorts results of measurements [22, 4 and 23].

In order to create defined fungal spore and bacterial concentrations in the suspensions, stability controls were carried out. The stability of the suspension over at least four hours is also important. Other studies recorded that the cultivable bacterial concentrations measured between 30 and 180 min remained stable and close to those measured during the first 30 min [24]. In their natural environment, bacteria often form aggregates such as microcolonies or biofilms [25]. In aerosol chamber testing, aggregates of microorganisms can form in the suspension. Therefore, the suspension should be kept as homogenous as possible for the aerosolization. The shear forces during aerosolization can damage the microorganisms which leads to a decrease of their concentration. This study agrees with Amato et al., 2015 that the aggregation seems to favor cell survival and that the transfer of cells from a liquid to the air is a critical step [26].

The stress during the impaction process affects the microbial recovery qualitatively and quantitatively [27]. In the active measurements of airborne microorganisms with the impaction method, more *A*. *niger* CFU/plate were counted than using the sedimentation method. Fungal spores do not seem as sensitive as bacteria and impacting them onto the agar probably does not cause them any appreciable harm. The number of *S*. *aureus* CFU/plate was clearly lower in the active method than in sedimentation. This is probably due to the collection stress to which gram-positive bacteria are exposed during air impaction by the impactor head and the subsequent impact on the agar. Bacterial cells can no longer proliferate which makes the number of CFU/plate lower than on the sedimentation plates where the tested strains are not exposed to such stress. Generally, spores and gram-positive bacteria are more robust than gram-negative bacteria and might survive in a harsh environment as well as tolerate more stress [28]. Other investigations also showed that the process of aerosolizing gram-negative bacteria causes a significant loss in bacterial viability [29]. However, Lin and Li observed a low level of total recovery of fungal spores collected in AGI-30 impingers, which may be due primarily to the higher biological stress during the sampling process [30].

For passive measurement of airborne microorganisms, different sampling times in the preliminary sedimentation tests were defined. The recommendation by Neumeister et al. to leave plates in the chamber exposed for at least one hour was confirmed by the present investigations [31]. In the passive measurements for *A*. *niger*, a variation in the sedimentation time between one and two hours did not show a significant difference in the number of CFU/plate. When measuring *S*. *aureus*, a sedimentation time of two hours, with the exception of two measurements, always yielded more CFU/plate than one hour. These findings might be explained by the fact that the loaded particles in the aerosol chamber may possibly form aggregates and remain suspended in the air for a long time or that all of the loaded material sank to the ground after only one hour. The particle size and the formation of aggregates and disaggregation can influence the settling time of different microorganisms [8, 32]. The literature shows that three hours after aerosolization of bacteria, the number of colony-forming units counted on a Petri dish was proportional to the number of living microorganisms suspended within the chamber [33]; the data of the present study for *S*. *aureus* did not support these findings.

The results of this study show correlations, parallels, and differences between the numbers of CFU obtained from impaction and sedimentation. Other studies reported a correlation between these two methods, although the tests were not standardized as opposed to the present study [8, 9]. However, a direct comparison between the two measuring systems is not possible because impaction and sedimentation are based on completely different measuring principles, which is emphasized in several studies [29, 3, 34 and 35].

The media for *A*. *niger* used in the present study yielded similar results. The number of CFU/plate increased continuously with increasing spore concentrations in the aerosol which led to R^2^ values for the two agar media of > 0.8 and r of > 0.9. That means that there is a very good linear relationship between the two measuring methods. As in several previous studies, the present experiment also found a correlation between the impaction and the sedimentation methods [8, 10 and 13]. The test series with *S*. *aureus* show deviations from the linear trend line at higher concentrations. This demonstrates that both measuring methods yield less precise results at higher concentrations. In the comparison between impaction and 1 h sedimentation, the low R^2^ can be explained by great differences in collection efficiency. The results show that after 1 h sedimentation, approximately 1000 CFU were counted on a plate, whereas the MAS-100NT® recovered only 250 CFU/plate, i.e. one-fourth. The comparison between impaction and 2 h sedimentation, the higher R^2^ of the sedimentation was due to the longer exposure time of the plates. The low r of 0.52 and 0.69 between 1 h sedimentation and impaction confirms, as does the R^2^, that a linear relationship barely exists.

With regard to the microbial recovery of the two measuring methods, there were differences in the case of *A*. *niger*. The ratio of CFU/plate between sedimentation and impaction of approximately 1:9.6 was probably due to the measuring volume which was very different in the two methods. With the active measuring method, 50 L of air each was impacted using the MAS-100NT®. By comparison, given a constant distribution of spores in the chamber and natural sedimentation without any disturbances such as air turbulences, one can calculate the approximate volume of a cylinder above a sedimentation plate. This resulted in a cylinder volume of 6 liters. Relative to the volume of one MAS measurement (50 L), this was a ratio of 1:8.3. The different collection volumes are a likely and logical explanation for the different recovery rates. Both the volumes and the results (CFU/plate) showed a very similar ratio which suggests that the two methods yield similar recovery rates when adjusted for the collection volume.

The recovery rate of *S*. *aureus* on CASO-agar showed a ratio of 1:1.96 between 1h and 2 h of sedimentation. This means that with only one hour more exposure time, almost twice as many microorganisms settled on the plates. There was a similar trend for CNA-agar (ratio of 1:1.91). Sedimentation measurements over a period of 4 h and overnight showed yet more CFU/plate on the agar. This fact supports the assumption that bacteria settle very slowly and that longer sedimentation periods are advantageous. Fykse et al. also indicated that within two hours of sampling the recovery rate showed a linear increase [27].

The comparison of impaction with 1 h and 2 h of sedimentation showed that passive sampling yielded 2.34- and 3.79-times as many CFU on the CASO plates. In contrast, the results for 1 h of sedimentation are lower on CNA-agar. Possibly, the almost identical number of CFU/plate (ratio of 1:0.97) of active and passive measurements of one hour is due to the selective CNA medium. The sedimentation of microorganisms seems to depend on a large number of factors such as: time, air flow, distribution of the microorganisms in the chamber, agar media, and position of plates in the aerosol chamber. In the case of the MAS-100NT®, the previously mentioned collection stress, which may lead to injury of the bacterial cells, is probably responsible for the fluctuating collection efficiency of the measuring device. The extent of injury that occurs in airborne microorganisms depends on the method of aerosol generation, the method of collection, the method of enumeration of aerosolized microorganisms [36–38].

The sedimentation method is less expensive and easier to conduct because it requires only an agar-containing Petri dish which is left open for a certain period of time [39]. According to Kramer et al., this method only provides approximate results and, in case of a very low concentration of microorganisms, the passive method may easily yield false negative results [40]. The sedimentation method can be used for qualitative investigations but it does not give accurate results of microorganism concentrations as the impaction method does.

## Conclusion

The active and passive collections of airborne microorganisms have advantages and disadvantages. The impaction requires a device for sample collection, therefore it is more expensive than the sedimentation method but facilitates the investigation of larger air volumes in a shorter period of time and is less likely to yield false negative results. This assertion, however, can only be made for the detection of fungal spores, especially *A*. *niger*, but not generally for microorganisms in general. The tests with *S*. *aureus* showed contrary results as the sedimentation method with a plate exposure time of 2 h showed higher CFU/plate counts than the impaction method with MAS-100NT® using a flow rate of 100 L/min. Sensitive bacteria are exposed to the suction, and then the impact on the agar leads to possible injury. This test series can be repeated using other species of bacteria or fungi and other measuring parameters.

## Supporting information

S1 Fig A and B. Statistical correction of the correlations.
(PDF)

S2 Fig Correlation of impaction and sedimentation of *A*. *niger* on MEA. (PDF)

S3 Fig Correlation of impaction and sedimentation of *A*. *niger* on DG18-agar. (PDF)

S4 Fig A—D. Correlation of impaction and sedimentation of *S*. *aureus* on CASO- and CNA-agar.
(PDF)
