# Correction: Comparative study of impaction and sedimentation in an aerosol chamber using defined fungal spore and bacterial concentrations

**DOI:** 10.1371/journal.pone.0197018

**Published:** 2018-05-03

**Authors:** Doris Haas, Herbert Galler, Carola Fritz, Christina Hasler, Juliana Habib, Franz F. Reinthaler

Fig 4 is incorrect. Please see the correct Fig 4 here.

**Fig 4:**
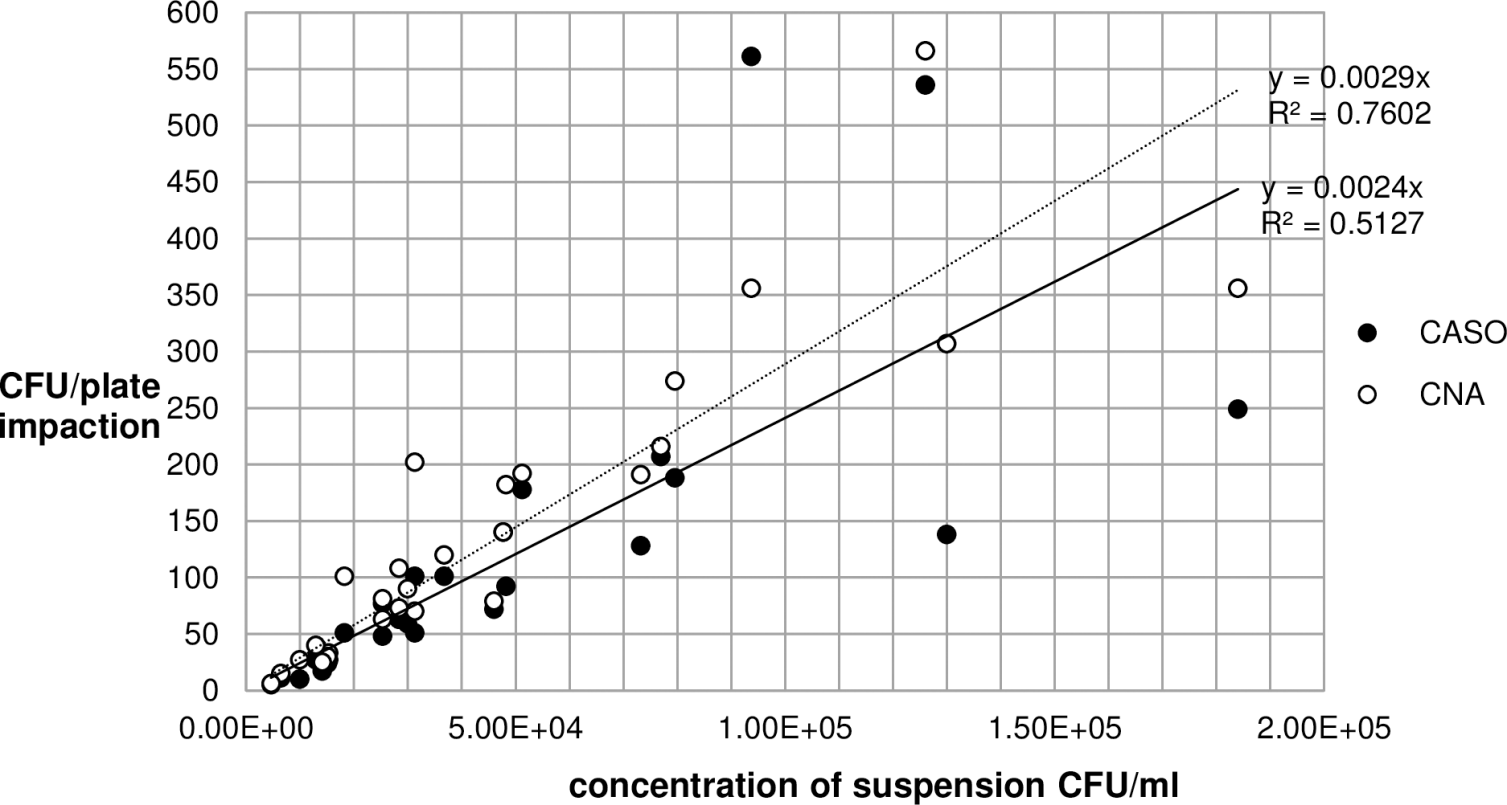
Comparison between CASO- and CNA-agar impaction for *S*. *aureus*.

## References

[1] Haas D, Galler H, Fritz C, Hasler C, Habib J, Reinthaler FF (2017) Comparative study of impaction and sedimentation in an aerosol chamber using defined fungal spore and bacterial concentrations. PLoS ONE 12(12): e0187039 https://doi.org/10.1371/journal.pone.0187039 29261663 10.1371/journal.pone.0187039 PMC5736173

