# Supplementary material for: Comparative study of impaction and sedimentation in an aerosol chamber using defined fungal spore and bacterial concentrations
Source: PLoS One. 2017 Dec 19;12(12):e0187039. doi: 10.1371/journal.pone.0187039 (PMC5736173; doi:10.1371/journal.pone.0187039)
Supplement: S1 Fig — A and B. Statistical correction of the correlations. (PDF) [file pone.0187039.s001.pdf]

S1 Figs. Statistical correction of the correlations

Figs A and B. The suction of 100 L air from the chamber by impaction method can lead to a decrease in the number of microorganisms in the repeated samples from the starting concentration of the suspension. To avoid the bias in the collecting data, a statistical correction for calculating the starting concentration of colony forming units (CFUs) was tested. For a proper renormalization, the exponential decay of the concentration has to be taken into account. This is achieved by replacing the exhausted volume V by the expression V0 [1-exp (-V/V0)], where V0 is the total volume of the chamber (glove box). In the vast majority of our measurements, V is much smaller than V0, which means that the above expression can be effectively replaced by V and the correction is of no relevance. As can be seen in Figure A and B, the corrected values remain within the error tolerances of our analysis. The error of the measurements is simply corrected by Poisson distribution of the data. The figures show that the corrections are within the error margin.

Fig. A Statistical correction of the correlation of impaction and sedimentation of *A. niger*

Fig. B Statistical correction of the correlation of impaction and sedimentation of *S. aureus*

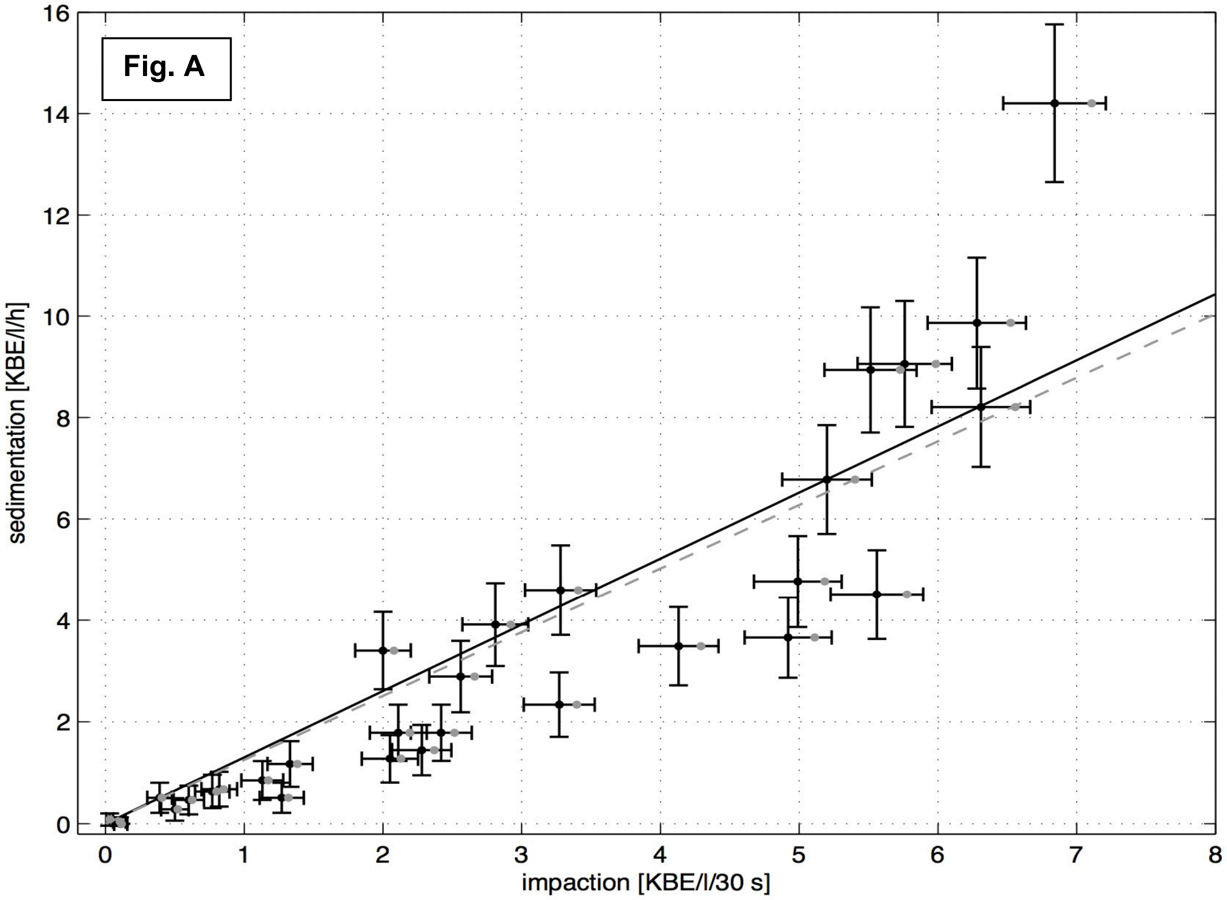
Fig. A
sedimentation [KBE/l/h]
impaction [KBE/l/30 s]
0
2
4
6
8
10
12
14
16
0
1
2
3
4
5
6
7
8

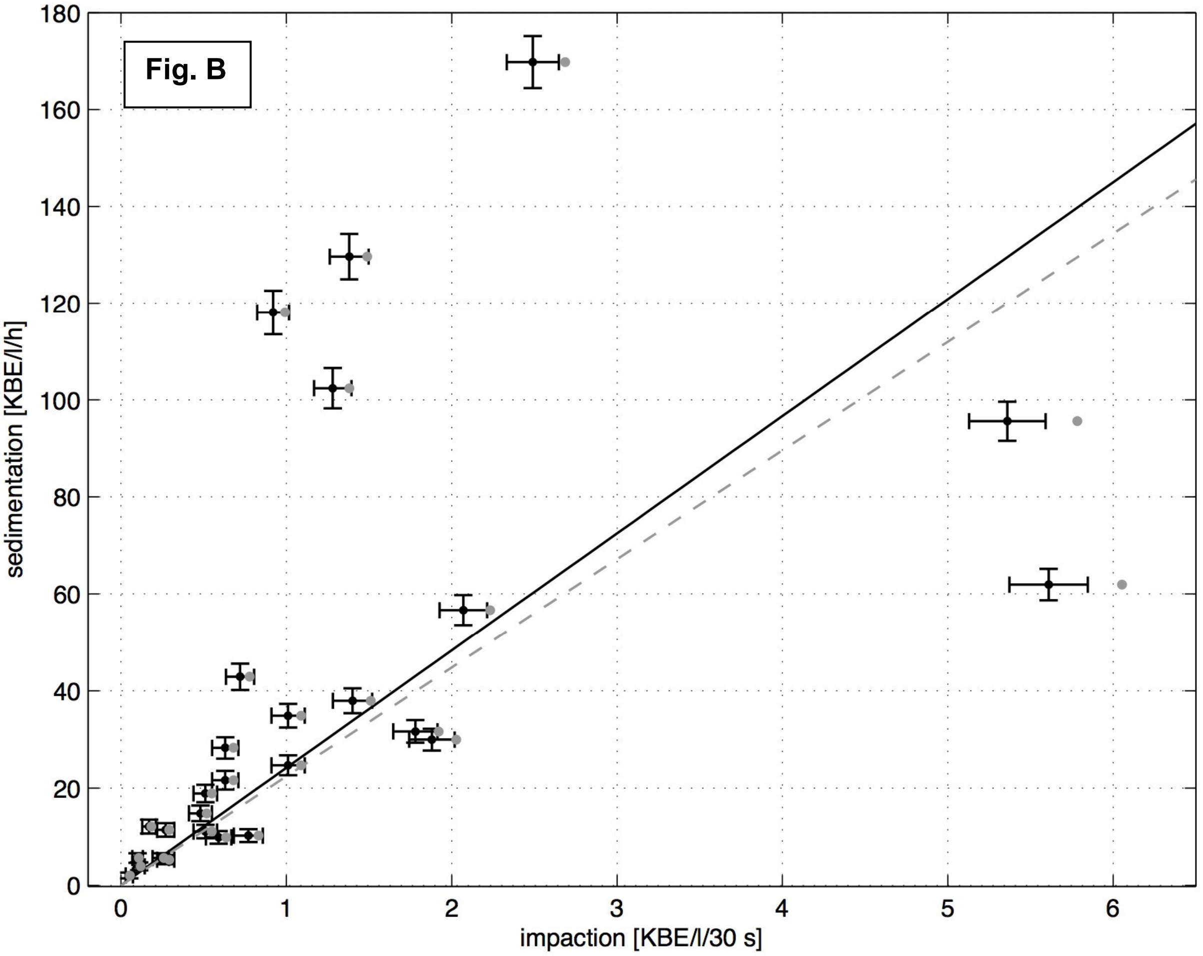
Fig. B
sedimentation [KBE/l/h]
impaction [KBE/l/30 s]
0
20
40
60
80
100
120
140
160
180
0
1
2
3
4
5
6
