# Supplementary figures and images for: Comparative study of impaction and sedimentation in an aerosol chamber using defined fungal spore and bacterial concentrations
Source: PLoS One. 2017 Dec 19;12(12):e0187039. doi: 10.1371/journal.pone.0187039 (PMC5736173; doi:10.1371/journal.pone.0187039)

1 **S2 Fig.** Correlation of impaction and sedimentation of *A. niger* on MEA

2

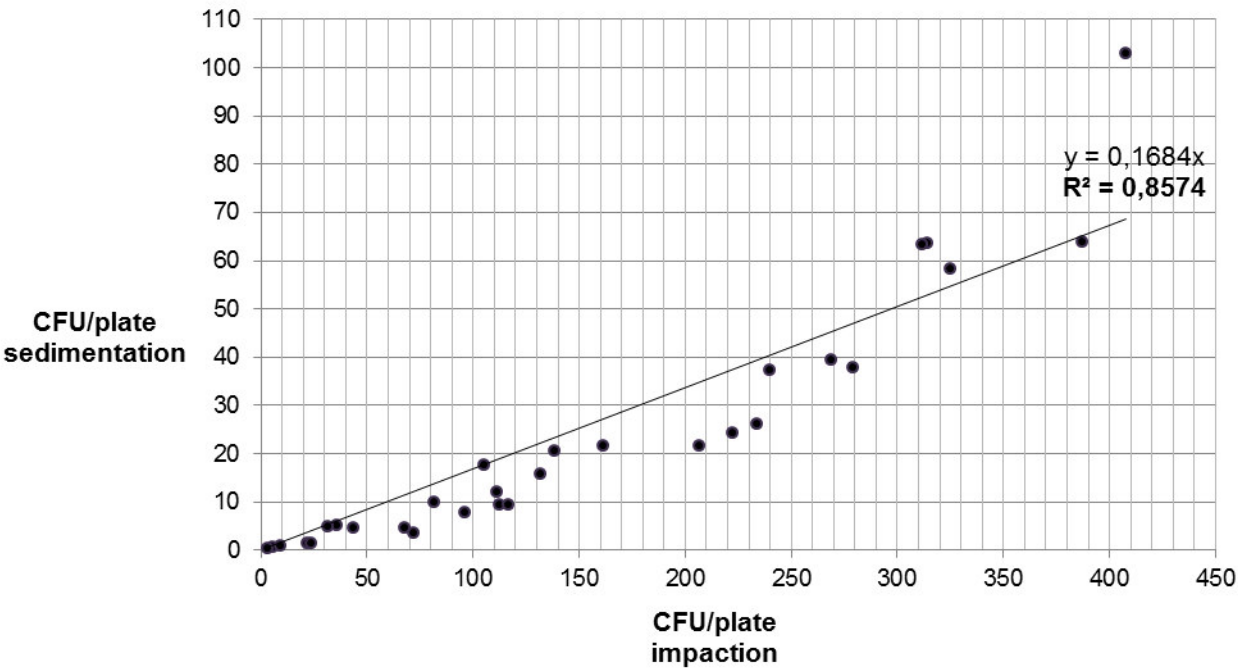

3

4

Supplement: S2 Fig — (PDF) [file pone.0187039.s002.pdf]

1 **S3 Fig.** Correlation of impaction and sedimentation of *A. niger* on DG18 agar

2

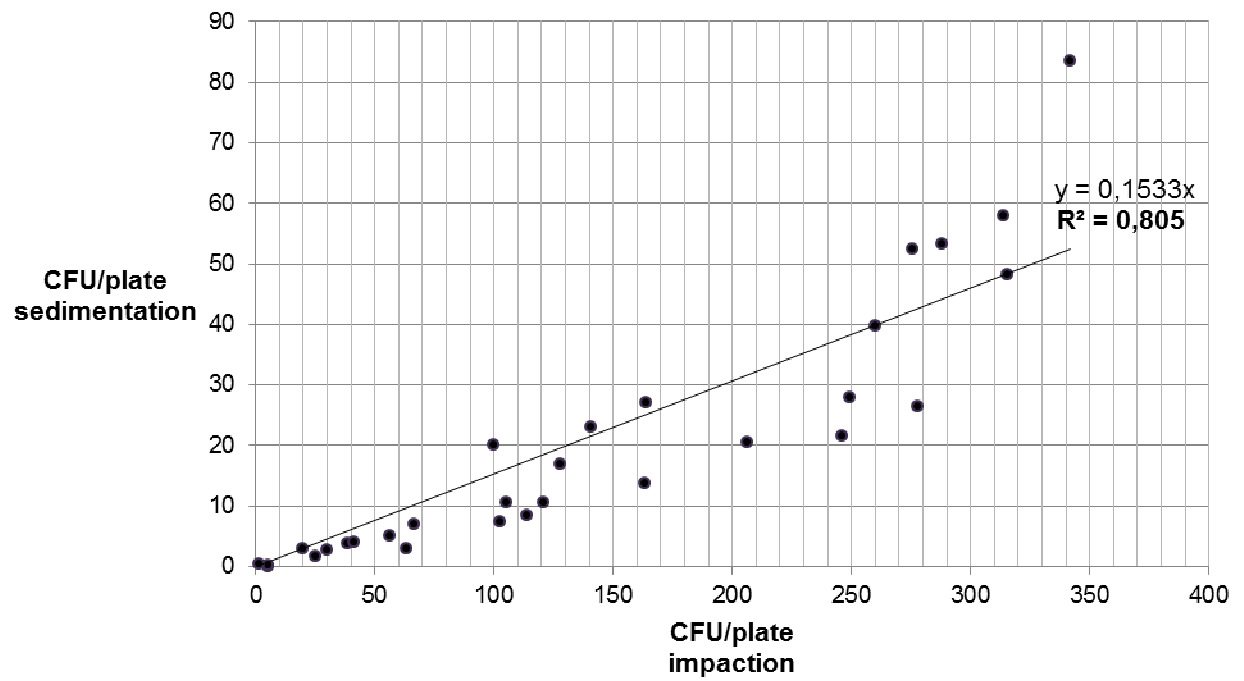

3

4

Supplement: S3 Fig — (PDF) [file pone.0187039.s003.pdf]
