# Supplementary material for: Comparative study of impaction and sedimentation in an aerosol chamber using defined fungal spore and bacterial concentrations
Source: PLoS One. 2017 Dec 19;12(12):e0187039. doi: 10.1371/journal.pone.0187039 (PMC5736173; doi:10.1371/journal.pone.0187039)
Supplement: S4 Fig — A—D. Correlation of impaction and sedimentation of S. aureus on CASO- and CNA-agar. (PDF) [file pone.0187039.s004.pdf]

S4 Figs. Correlation of impaction and sedimentation of *S. aureus* on CASO- and CNA-agar.

The correlation of impaction and 1 h and 2 h sedimentation of *S. aureus* on CASO-agar and CNA-agar show a linear correlation at low numbers of CFU/plate. After two hours sedimentation R^2 values were higher that one hour sedimentation.

Figs A and B. Correlation of impaction and sedimentation of *S. aureus* 1 h and 2 h on CASO-agar.

Figs C and D. Correlation of impaction and sedimentation of *S. aureus* 1 h and 2 h on CNA-agar.

Fig. A

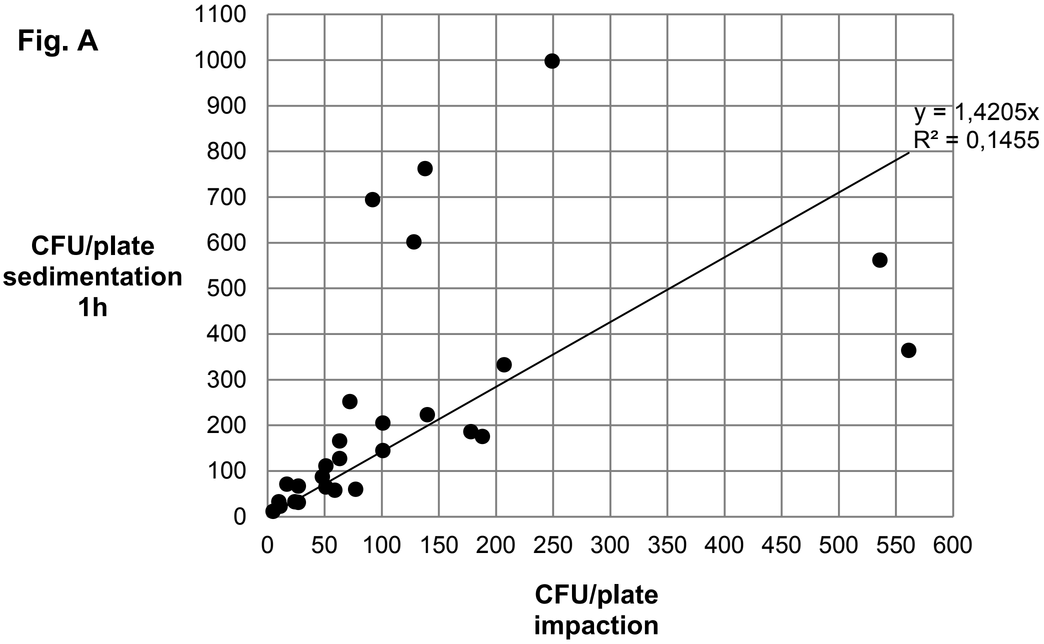

20

Fig. B

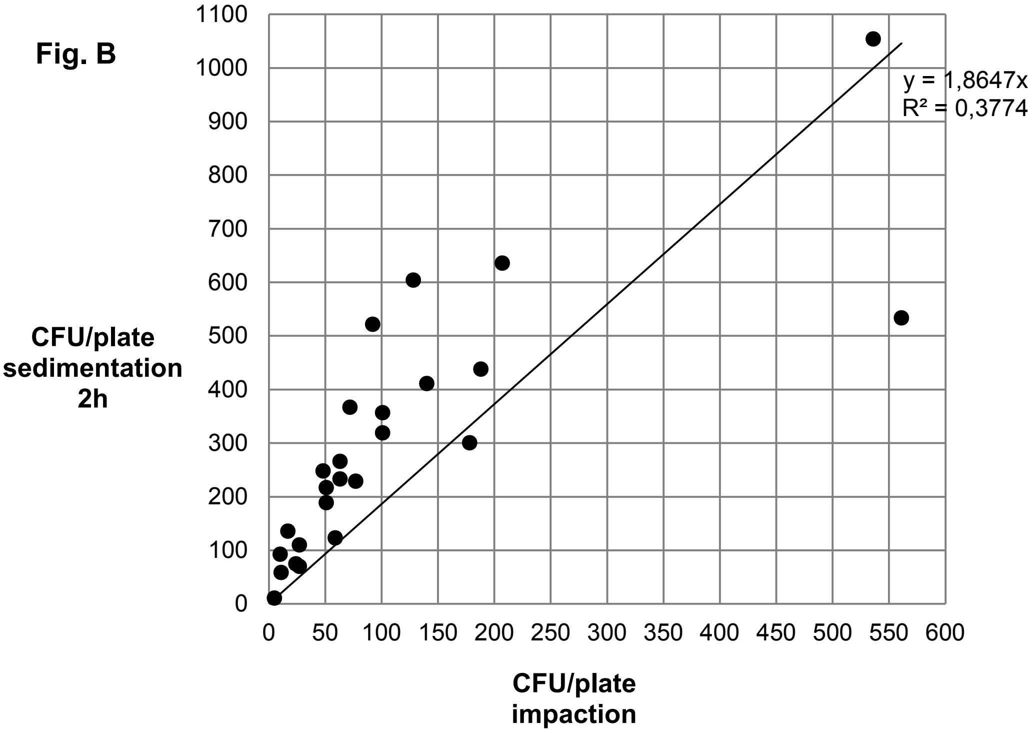

21

Fig. C

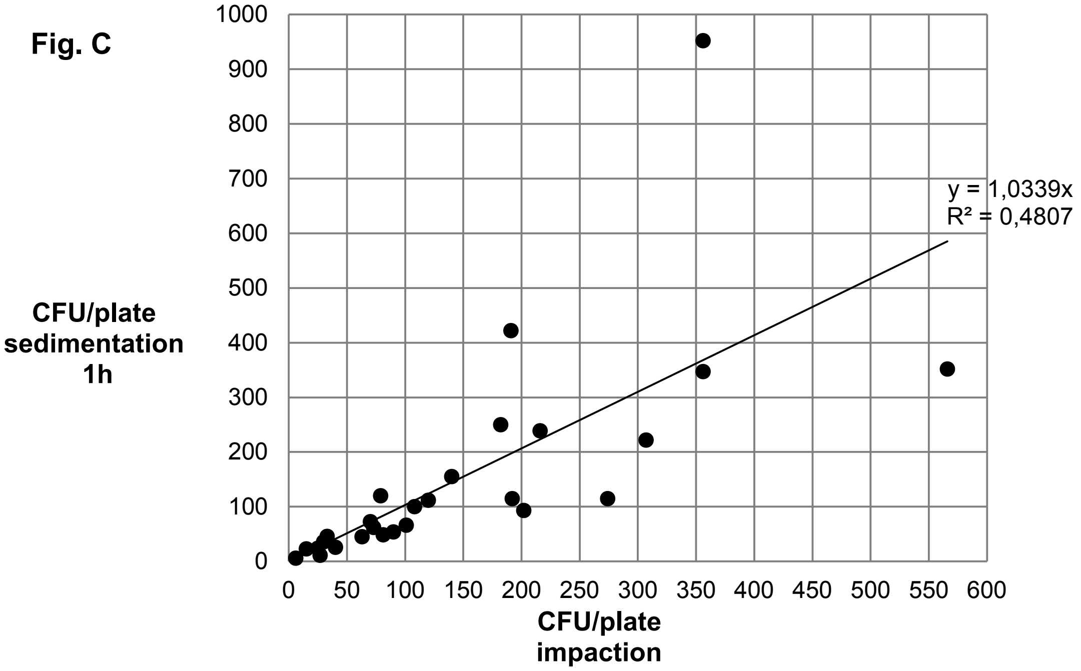

1000
900
800
700
600
500
400
300
200
100
0
CFU/plate sedimentation 1h
0 50 100 150 200 250 300 350 400 450 500 550 600
CFU/plate impaction
y = 1,0339x
R² = 0,4807

Fig. D

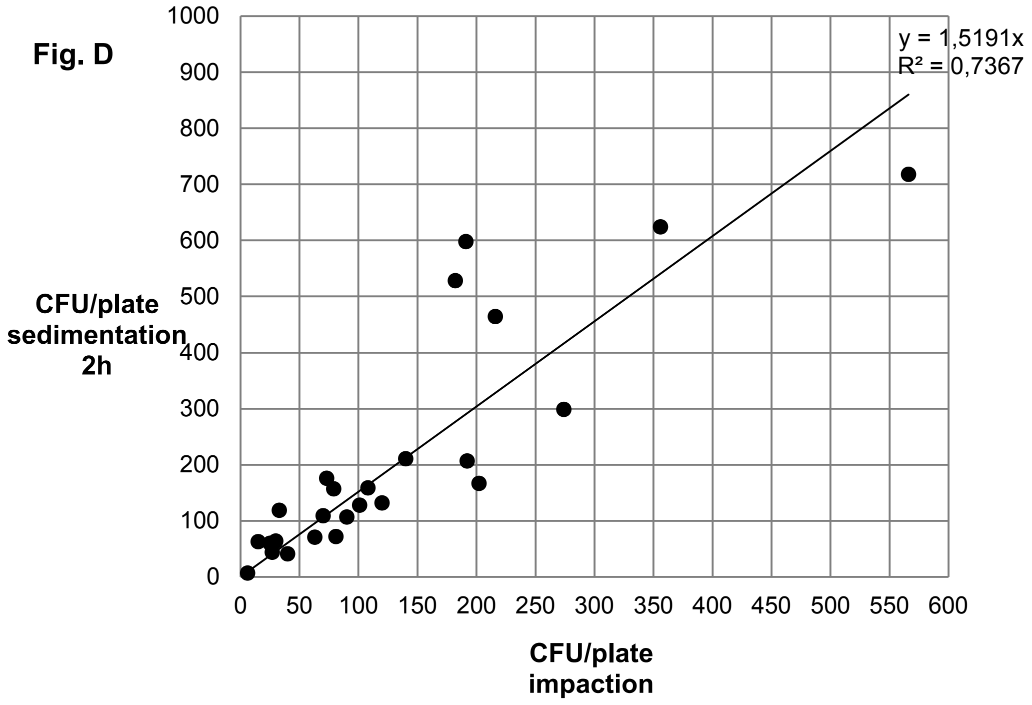

1000
900
800
700
600
500
400
300
200
100
0
CFU/plate sedimentation 2h
0 50 100 150 200 250 300 350 400 450 500 550 600
CFU/plate impaction
y = 1,5191x
R² = 0,7367
